# Endogenous Asymmetric Dimethylarginine Pathway in High Altitude Adapted Yaks

**DOI:** 10.1155/2015/196904

**Published:** 2015-08-26

**Authors:** Shiro Mizuno, Takeshi Ishizaki, Hirohisa Toga, Akio Sakai, Jainagul Isakova, Elnura Taalaibekova, Zamirbek Baiserkeev, Baktybek Kojonazarov, Almaz Aldashev

**Affiliations:** ^1^Department of Respiratory Medicine, Kanazawa Medical University, Ishikawa 920-0293, Japan; ^2^Department of Health Science, Matsumoto University, Matsumoto 390-1295, Japan; ^3^Laboratory of Molecular and Cell Biology, Institute of Molecular Biology and Medicine, 720040 Bishkek, Kyrgyzstan; ^4^Excellence Cluster Cardio-Pulmonary System, Universities of Giessen & Marburg Lung Center, 35392 Giessen, Germany

## Abstract

Hypoxia-induced and high altitude pulmonary hypertension are a major problem in the mountain areas of the world. The asymmetric methylarginines (ADMA) inhibit nitric oxide (NO) synthesis by competing with L-arginine, and high levels of plasma ADMA predict adverse outcomes in pulmonary hypertension. However, little is known about the regulation of the ADMA-NO pathway in animals adapted to high altitudes. We measured the plasma ADMA concentration, endothelial NO synthase (eNOS), dimethylarginine dimethylaminohydrolases (DDAH) protein expression, and DDAH activities in the lungs from yaks. Although the yaks are hypoxemic, cardiac function and pulmonary arterial pressures are almost normal, and we found decreased DDAH expression and activity in association with reduced plasma ADMA concentrations. The eNOS expression was significantly higher in yaks. These results indicate that augmented endogenous NO activity in yaks through the ADMA-DDAH pathway and eNOS upregulation account for the low pulmonary vascular tone observed in high altitude adapted yaks.

## 1. Introduction

It is well known that endogenous nitric oxide (NO) plays a pivotal role in maintaining the low pulmonary vascular tone patients with pulmonary hypertension [1]. Recent studies imply that asymmetric dimethylarginine (ADMA), an endogenous guanido-substituted analogue of L-arginine, also plays a critical role as a natural inhibitor of nitric oxide synthase (NOS) in the regulation of endogenous NO production. ADMA is synthesized* via* the methylation of protein arginine residues by the enzyme protein arginine methyltransferase 1 (PRMT1) [2] and competes with L-arginine for NOS and inhibits NO formation [3]. ADMA is mainly inactivated by dimethylarginine dimethylaminohydrolases (DDAH) by hydrolysis to L-citrulline. Because ADMA is degraded by DDAH, the plasma and tissue levels of ADMA are thought to be regulated by the DDAH activities. Previous studies showed that the overexpression of DDAH reduced tissue and plasma levels of ADMA which was associated with an increased production of NO, a reduction in systemic vascular resistance, and reduced atherosclerosis in transgenic mice [4, 5]. The DDAH activity also has a protective role in insulin resistance [6, 7] and progression of chronic kidney disease, which are both associated with reduced level of ADMA and increased endogenous NO production [8, 9]. A reduced expression or pharmacological inhibition of DDAH results in elevated levels of ADMA associated with decreased reaction of NO-mediated vasodilation [10] which supports the idea that local tissue DDAH activity plays an important role in the maintenance of vascular tone and NOS activity* via* ADMA production. Notably, the plasma level of ADMA not only is a simple biomarker of many diseases, but also could be of functional importance and a key substance, especially in the progression of cardiovascular and metabolic diseases associated with endothelial dysfunction [11, 12]. Increased plasma levels of ADMA may contribute to impaired NO synthesis leading to atherosclerosis, progression of cardiovascular disease, diabetes mellitus, and chronic renal disease [13, 14]. A functional role of plasma levels of ADMA has been attributed to endothelial cell dysfunction in pulmonary hypertension [15–20], cardiovascular disease, chronic renal disease, and portal hypertension in liver cirrhosis [21]. In patients with pulmonary hypertension, elevated levels of ADM are closely correlated with decreased exercise tolerance [19], the severity of pulmonary hypertension, and a poor prognosis [15, 18].

Pulmonary hypertension is a common complication among highlanders and animals that live at high altitudes. Chronic exposure to a decreased oxygen pressure causes chronic hypoxemia and secondary pulmonary hypertension and pulmonary vascular remodeling in human and animals [22, 23]. After several weeks of exposure to high altitude, lowlanders develop pulmonary hypertension, which is not completely reversed by supplemental oxygen [24], suggesting development of vascular remodeling of the lung [25]; this vascular remodeling is also related to a decreased activity of NOS [26, 27]. However, high altitude adapted animals, such as yak, llama, and the Tibetan sheep, have low pulmonary vascular tone, normal right ventricular function, and also a decreased hypoxic pulmonary vasoconstriction response [28–31]. We previously reported an enhanced effect of NOS inhibition in the yak pulmonary circulation [32], consistent with an augmented production of NO in yaks living at high altitude.

Because of these previous findings, we hypothesized that elevated activity of DDAH in the lung tissue, which suppresses ADMA expression, could be a regulatory factor of the pulmonary vascular tone in yaks. In order to address this hypothesis, we assessed eNOS and DDAH protein expression and activity of DDAH of lung tissues obtained from yaks living at high altitude and we also measured nitrites and ADMA concentrations in the yak plasma.

## 2. Materials and Methods

### 2.1. Animals

Young (2 years old) male yaks with an estimated weight of 200 kg were used for the experiment. Yaks were living at Archaly (2900–3000 m above sea level). Male bovines (2 years old) with an estimated weight of 200 kg were used as a control group. The cattle were living in Bishkek (760 m above sea level).

### 2.2. Hemodynamic Studies

A balloon-tipped pulmonary arterial catheter was inserted percutaneously into a right internal jugular vein and advanced to the pulmonary artery for measurement of pulmonary artery pressure (РРА) and pulmonary capillary wedge pressure (PWP). A plastic catheter was placed percutaneously into the right internal jugular artery to monitor the systemic arterial pressure and heart rate (HR). Arterial oxygen saturation (SaO_2_) was measured by pulse oximetry. The cardiac output (CO) was measured by the thermodilution method using a Swan-Ganz catheter and a cardiac output computer (Vigilance).

### 2.3. Western Blot Analysis

Lung protein extracts were prepared for Western blot analysis by homogenization of tissue samples and the protein concentration was determined by the Bradford method (BioRad). Homogenates (100 *μ*g for eNOS, 20 *μ*g for DDAH I, or 50 *μ*g for DDAH II) were separated by SDS-PAGE (7.5% for eNOS, 12% for DDAH I and DDAH II) and then transferred to nitrocellulose membranes (BioRad). Blots were blocked with TBS buffer (50 mmol/L Tris-HCl, pH 7.4, 0.15 mol/L NaCl, 0.1% Tween-20) plus 2% (wt/vol) BSA or 5% (wt/vol) nonfat milk for 1 hour at room temperature and then incubated for 1 hour at room temperature with an eNOS monoclonal antibody (1 : 500 dilution in TBS plus BSA, Transduction Laboratories), a DDAH I monoclonal antibody, or a DDAH II polyclonal antibody. Immunoreactive proteins were detected following incubation with a peroxidase-conjugated antibody and enhanced chemiluminescence (Amersham). The relative protein expression was quantified by densitometric analysis.

### 2.4. Measurement of DDAH Activity

Freshly prepared lung tissue of yaks and cows was homogenized with sodium phosphate buffer, pH 6.5, at 4°C in a glass homogenizer. The homogenate was centrifuged at 10,000 g for 30 min to obtain the supernatant. Briefly, the lysate was incubated with 4 mmol/L ADMA and 0.1 mol/L sodium phosphate buffer (pH 6.5) in a total volume of 0.5 mL for 2 hours at 37°C. The reaction was stopped by the addition of an equal volume of 10% trichloroacetic acid, and the supernatant was boiled with diacetyl monoxime (0.8% in 5% acetic acid) and antipyrine (0.5% in 50% sulfuric acid). The amounts of L-citrulline formed were determined by spectrophotometric analysis at 466 nm. As the assay blank, the enzyme preparations heated in a boiling water bath were subjected.

### 2.5. Measurement of Plasma Nitrite, ADMA, and Endothelin-1 (ET-1) Concentration

The concentrations of plasma ADMA were measured using an ELISA immunoassay kit according to the manufacture's protocol (DLD Diagnostika Gmbh, Germany). The nitrite concentration in plasma was measured using the Griess reagent system from Promega (USA), and the plasma concentrations of ET-1 were determined by high power liquid chromatography (HPLC).

### 2.6. Statistical Analysis

The results are expressed as mean ± SD. The statistical analysis was performed using Student's *t*-test. Comparisons were considered statistically significant at *P* < 0.05.

## 3. Results

### 3.1. Parameters of Pulmonary Hemodynamics in High Altitudes Yaks

Pulmonary hemodynamic data in high altitudes yaks are summarized in Table 1. Although the arterial oxygen saturation of yaks is apparently below normal, the pulmonary hemodynamics including the mean pulmonary arterial pressure and the PVR were almost normal.

### 3.2. Nitrates, ADMA, and ET-1 Concentrations in Yaks and Bovines

**Figure 1:**
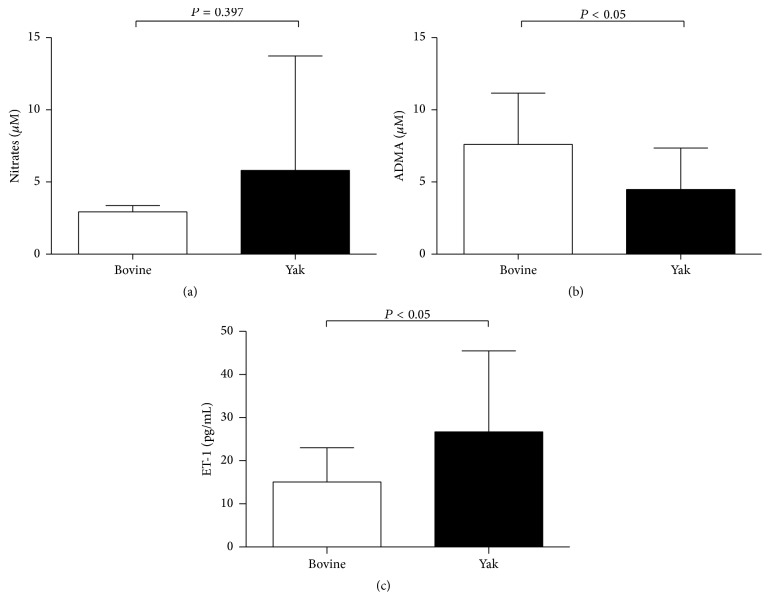
Nitrates, ADMA, and endothelin-1 (ET-1) concentrations of plasma from yaks and bovines. Nitrate concentration, measured by Griess method, showed no significant differences between yaks (*n* = 12) and bovines (*n* = 6) (a). ADMA concentration, measured by ELISA, was significantly lower in yaks (*n* = 19) than bovines (*n* = 11) (b), and ET-1 concentration was significantly higher in yaks (*n* = 13) than bovines (*n* = 10) (c). Data are expressed as mean ± SD. ^*∗*^ *P* < 0.05 versus bovine.

Although the average nitrate concentration is slightly higher in yaks when compared to that in the bovine, there were no significant differences in serum nitrates concentrations between bovines and yaks. The serum levels of ET-1 in yaks were significantly increased compared with that from bovines, and the serum ADMA levels were significantly decreased compared with that from bovines (Figures 1(a), 1(b) and 1(c)).

### 3.3. eNOS, DDAH Protein Expression, and DDAH Enzyme Activity

**Figure 2:**
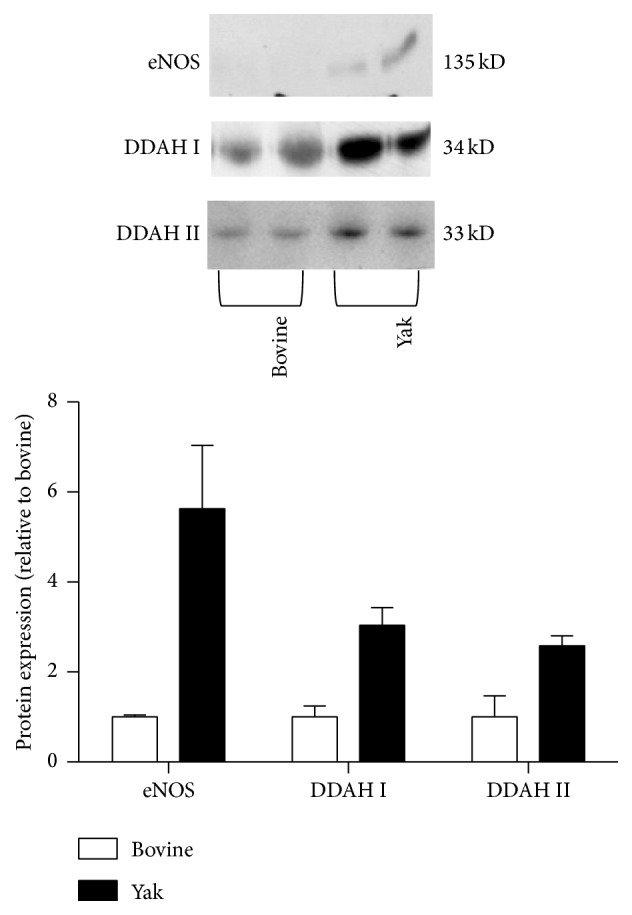
Western blot analysis of eNOS, DDAH I, and DDAH II protein in lungs from yaks and bovines. The photomicrograph shown is a representative image from the experiments, and the bar graph shows the density ratios of eNOS, DDAH I, and DDAH II protein bands relative to those from bovines. The eNOS, DDAH I, and DDAH II protein expression was apparently increased in lungs from yaks compared with those from bovines. Data are expressed as mean ± SD (*n* = 2).

The Western blot analysis of lung homogenates showed that the expression of the eNOS protein in the lungs from yaks was markedly higher when compared with that from bovines, and both the DDAH I and DDAH II protein expression were apparently increased in the lungs from yaks when compared with those from bovines (Figure 2).

**Figure 3:**
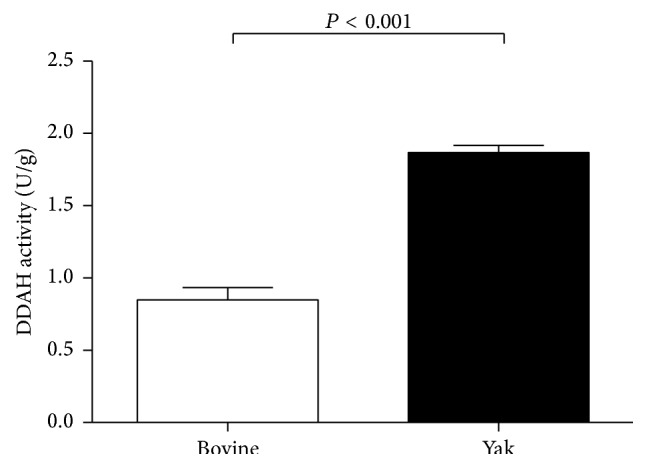
DDAH activity in lungs from yaks and bovines. The bar graph shows DDAH I activity in the lungs from yaks and bovines. The DDAH activity is significantly increased in lungs from yaks compared with those from bovines. Data are expressed as mean ± SD (*n* = 6). ^*∗*^ *P* < 0.05 versus bovine.

The protein DDAH expression and the DDAH enzyme activity in the lungs from yaks were significantly higher, roughly twice, as high when compared with the bovine samples (Figure 3).

## 4. Discussion

Our present experiments confirmed our previous studies showing augmented NO activity in yaks living at high altitudes. We had previously inferred this result from data obtained following the administration of the NOS inhibitor, N^w^-nitro-L-arginine (NLA) [32]. We now find increased DDAH expression and activity associated with low levels of plasma ADMA in yaks. The increased DDAH protein expression and DDAH activity in the lungs from yaks is in line with previous findings showing regulatory effects of DDAH expression in regard to the NO-ADMA-DDHA pathway [33]. Impaired DDAH activity results in the accumulation of ADMA in animals [34]. Our results of increased expression and activity of DDAH in the lungs from yak raise the possibility that the low pulmonary vascular tone in yaks living at high altitudes is derived from enhanced NO production and NOS activity due to decreased expression of the endogenous NOS inhibitor in the lung tissue. In addition, our findings of decreased levels of ADMA in plasma from yaks support a role of ADMA as a biomarker of pulmonary pressure regulation [15, 35].

The lungs are thought to be a major source of NOS and ADMA [36]. Many previous studies have shown increased plasma levels of ADMA in patients with various forms of PAH, including idiopathic PAH [15, 16], PAH associated with chronic thromboembolism [18], sickle cell disease-related PH [20, 37], PAH associated with collagen vascular disease [19], and congenital heart disease [17]. It is possible that shear stress-induced PRMT activity [38] and downregulation of DDAH induced by alveolar hypoxia [39] could be responsible for the increased plasma ADMA expression. However, because of the decreased SaO_2_ and the normal pulmonary arterial pressure of the yaks, we cannot explain the increased expression of DDAH in our study as caused by pulmonary vascular shear stress in the yaks. At present, the precise mechanism of high DDAH expression in yaks remains uncertain, and we speculate that an evolutionary process of yaks living at high altitudes has altered the regulation of the ADMA-DDAH pathway in such a way as to provide protection against PAH* via* augmented local activation of NO production in the lungs.

Previous results showing decreased eNOS expression in PAH patients and increased levels of ADMA [18] suggest that circulating ADMA is a potential negative regulator of eNOS expression in pulmonary arteries. Although it has not been investigated whether circulating ADMA influences eNOS expression, the inhibitory effect of ADMA in the pulmonary vascular tone may be attenuated by the increased NOS protein expression as we found in our yak study. There is evidence that systemic infusion of ADMA also increases the systemic vascular resistance and decreases the cardiac output in humans [40] and this finding supports the role of plasma ADMA levels in the pulmonary circulation of yaks as an adaptive mechanism.

Despite the lower ADMA plasma levels and increased eNOS expression in yaks, we could not find significant differences in nitrate concentrations between yaks and bovines (Figure 1(a)). This discrepancy may be explained by the decreased oxygen concentration in the lungs and perhaps by different diets of the two animal species. Our result showing higher levels of ET-1 in yaks is consistent with an alveolar hypoxia- or hypoxemia-induced activation of hypoxia-induced factor alpha (HIF-1*α*) and ET-1 pathways in the yaks [41]. Because oxygen is necessary for NO production by NOS, alveolar hypoxia will result in a reduced activity of NOS [11]. In addition, it is known that exogenous food-derived nitrate could also affect plasma nitrate levels, in addition to the L-arginine-NO pathway [1].

In conclusion, the results of this study show augmented expression of DDAH expression and activity associated with reduced levels of plasma ADMA in yaks. The results of augmented activity of NOS in yaks could be explained by the ADMA-DDAH-NO pathway. However, further investigation regarding the plasma ADMA and DDAH activity of the adapted animals and/or humans living at high altitudes is necessary to determine whether decreased plasma ADMA levels impact the eNOS expression in the lung and the pulmonary arterial pressure.

## Figures and Tables

**Table 1:** Pulmonary hemodynamics in yaks.

PAP mean, mm Hg	18.5 ± 0.7
PAP systolic, mm Hg	25 ± 1.4
PWP, mm Hg	8.5 ± 0.7
CO, L/min	10.5 ± 0.7
PVR, dyne·sec·cm^−5^	76 ± 5.6
HR, b/min	59 ± 1.4
SaO_2_, %	87 ± 1.4

PAP: pulmonary arterial pressure, PWP: pulmonary capillary wedge pressure, CO: cardiac output, PVR: Pulmonary Vascular Resistance, HR: heart rate, and SaO_2_: arterial oxygen saturation. Data are expressed as mean ± SD (*n* = 3).
